# Transcriptomic analysis of the zebrafish inner ear points to growth hormone mediated regeneration following acoustic trauma

**DOI:** 10.1186/1471-2202-12-88

**Published:** 2011-09-02

**Authors:** Julie B Schuck, Huifang Sun, W Todd Penberthy, Nigel GF Cooper, Xiaohong Li, Michael E Smith

**Affiliations:** 1 Department of Biology, Western Kentucky University, 1906 College Heights Blvd., Bowling Green, KY 42101, USA; 2 Department of Molecular Biology and Microbiology, Burnett School of Biomedical Science, University of Central Florida College of Medicine, 4000 Central Florida Blvd., Orlando, FL 32816, USA; 3 Department of Anatomical Sciences & Neurobiology University of Louisville, School of Medicine, 500 South Preston Street, Louisville, KY 40202, USA

## Abstract

**Background:**

Unlike mammals, teleost fishes are capable of regenerating sensory inner ear hair cells that have been lost following acoustic or ototoxic trauma. Previous work indicated that immediately following sound exposure, zebrafish saccules exhibit significant hair cell loss that recovers to pre-treatment levels within 14 days. Following acoustic trauma in the zebrafish inner ear, we used microarray analysis to identify genes involved in inner ear repair following acoustic exposure. Additionally, we investigated the effect of growth hormone (GH) on cell proliferation in control zebrafish utricles and saccules, since GH was significantly up-regulated following acoustic trauma.

**Results:**

Microarray analysis, validated with the aid of quantitative real-time PCR, revealed several genes that were highly regulated during the process of regeneration in the zebrafish inner ear. Genes that had fold changes of ≥ 1.4 and *P *-values ≤ 0.05 were considered significantly regulated and were used for subsequent analysis. Categories of biological function that were significantly regulated included cancer, cellular growth and proliferation, and inflammation. Of particular significance, a greater than 64-fold increase in growth hormone (*gh1*) transcripts occurred, peaking at 2 days post-sound exposure (dpse) and decreasing to approximately 5.5-fold by 4 dpse. Pathway Analysis software was used to reveal networks of regulated genes and showed how GH affected these networks. Subsequent experiments showed that intraperitoneal injection of salmon growth hormone significantly increased cell proliferation in the zebrafish inner ear. Many other gene transcripts were also differentially regulated, including heavy and light chain myosin transcripts, both of which were down-regulated following sound exposure, and major histocompatability class I and II genes, several of which were significantly regulated on 2 dpse.

**Conclusions:**

Transcripts for GH, MHC Class I and II genes, and heavy- and light-chain myosins, as well as many others genes, were differentially regulated in the zebrafish inner ear following overexposure to sound. GH injection increased cell proliferation in the inner ear of non-sound-exposed zebrafish, suggesting that GH could play an important role in sensory hair cell regeneration in the teleost ear.

## Background

Deafness is a widespread problem with tremendous societal costs, but effective treatments for hearing loss have remained elusive. Therapeutics that can successfully treat or prevent the onset of deafness are desperately needed. To develop such treatments, a thorough understanding of the process of auditory hair cell death and regeneration must be established. Mammalian cochlear hair cells do not regenerate after they have been destroyed, and vestibular hair cells show limited capacity to regenerate [1]. However, avian and teleost sensory hair cells regenerate [2-4] through direct trans-differentiation [5-9] or mitosis [10-16]. The genes responsible for conferring teleost regenerative capacity are unknown; however, most of the zebrafish genome has been sequenced and resources are available for the identification of gene function [17]. The mechanosensory hair cell of teleosts resembles that of the human hair cell at both the structural and functional level [18], and there is a high degree of evolutionary conservation of chromosomal synteny extending from zebrafish to human [19,20]. Mammals share homologous genes with fish that are known to affect inner ear structure and/or function. For instance, the zebrafish *Mariner *mutant possesses a missense mutation in the gene encoding myosin VIIA and presents functional and morphological hair cell defects that are similar to those found in mice defective in Myosin VIIA [21]. *Foxi1*, a gene expressed in otic precursor cells, is necessary for normal inner ear development in both mice [22] and zebrafish [23]. *Atoh1 *(atonal homolog 1, previously *Math1*) is required for differentiation of hair cells in rodents [24,25] while a similar role is carried out by zebrafish homologs *atoh1a *and *atoh1b *[26]. Since zebrafish share inner ear developmental and differentiation genes with mammals, examination of gene expression in the zebrafish during hair cell regeneration may uncover new targets for genetic manipulation leading to hair cell regeneration in mammals.

Investigators have induced auditory hair cells to proliferate in postnatal mammals using gene therapies that disrupt the normal pathways that keep mammalian cochlear hair cells and their surrounding supporting cells in a terminally differentiated state. The cyclin-dependent kinase inhibitor p27^Kip1 ^(Cdkn1b), the tumor suppressor retinoblastoma protein (Rb), and transcription factor Atoh1 have been investigated as potential therapeutic targets [27-30]. To date, gene manipulation studies have proven unsuccessful in producing auditory hair cells of the proper quantity and arrangement [27], maturity and function [31], or location [32]. Adjusting the timing and/or sequence of manipulation of the above-mentioned targets may produce more satisfactory results; however, other targets that have not yet been identified may prove to be key regulators of auditory hair cell regeneration.

We have recently established a basic time line of sound-induced cell proliferation and hair cell bundle recovery in the zebrafish saccule following acoustically-induced damage [33]. By performing zebrafish-based transcriptomic analysis following acoustic overexposure, the purpose of the current study was to identify genes that are important in the recovery and regeneration of teleost, and perhaps mammalian, hair cells. Such gene pathway analyses may help identify potential targets for therapeutic intervention. In this study, we report on the role of growth hormone-mediated signaling in hair cell proliferation and present a number of other genes differentially regulated following acoustic overstimulation, including those for major histocompatibility proteins and myosins.

## Results

### Comparative transcriptome analysis of time points following acoustic overexposure

We were interested in the changes in gene regulation that occurred on and between 2 and 4 days post-sound exposure (dpse) to a 100 Hz tone at 179 dB re 1 μPa Root Mean Squared (RMS) for 36 h, as previous work indicated that this level of sound exposure produced significant hair cell damage in the zebrafish saccule. In this previous study, hair cell damage was followed by significant cell proliferation that peaked at 2 dpse and fell to control levels before 4 dpse. Additionally, hair cell bundle density on the saccular macula decreased immediately following acoustic exposure and then increased between 2 and 7 dpse, indicating that hair cell replacement and/or repair took place during this time interval [33]. We hoped to detect differential expression of genes involved in zebrafish auditory hair cell replacement and/or repair during this time period.

**Figure 1:**
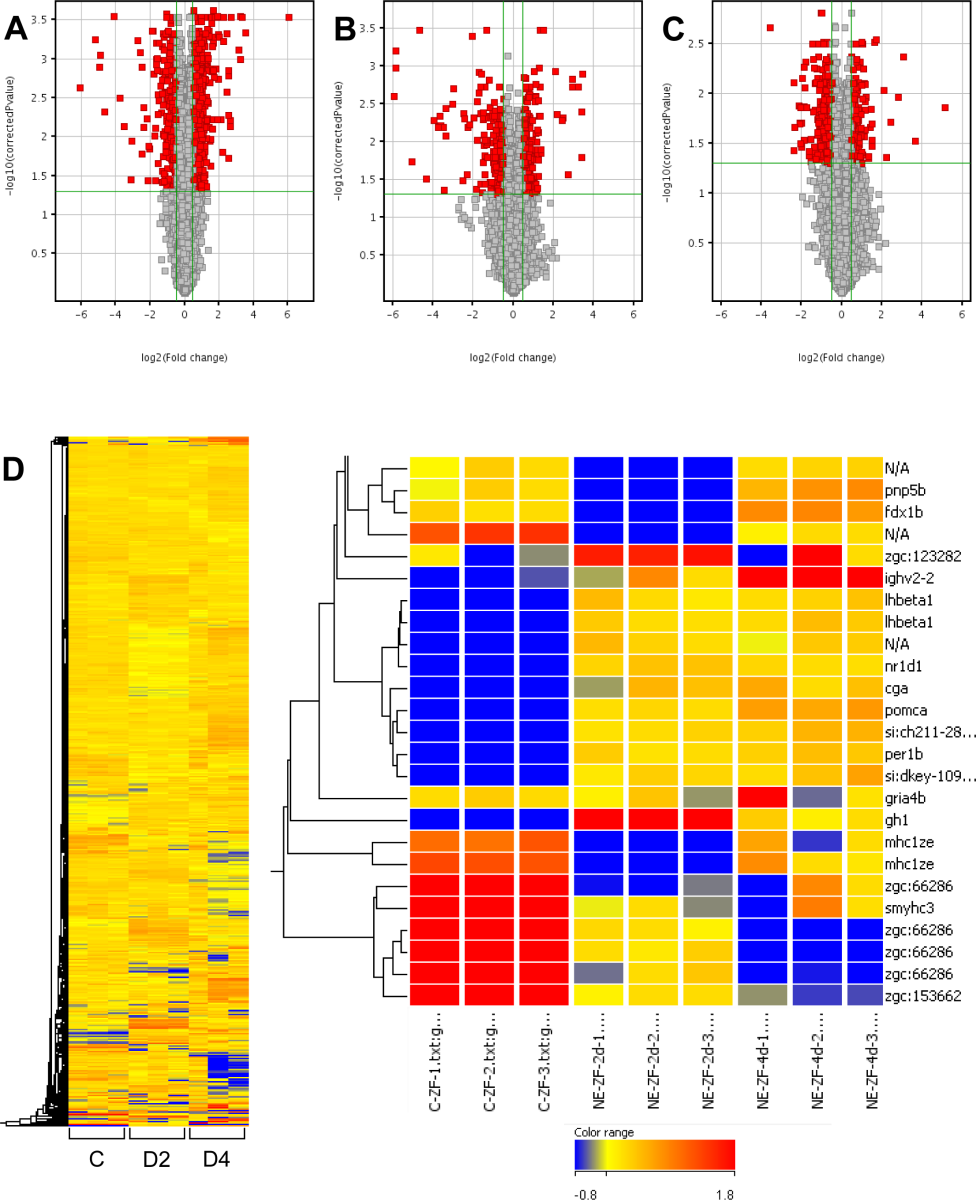
**Volcano plots and heat maps showing the time course of acoustic trauma-related differential gene expression in the zebrafish inner ear**. RNA samples from zebrafish inner ears of controls or subjects exposed to acoustic trauma and allowed to recover for either two or four days and then hybridized to a zebrafish microarray, which measures expression (mRNA abundance) for 21,000 gene transcipts. Volcano plots show differential gene expression between (A) controls and two days post-trauma (D2), (B) controls and four days post-trauma (D4), and (C) two versus four days post-trauma. The Y-axes of the volcano plots display the negative log (base 10) of *P *values from paired *t*-tests, while the X-axes show the log (base 2) of the fold differences between the groups. The horizontal line represents the *P *= 0.05 value, and the vertical lines corresponding to fold differences of 1.4 and -1.4, respectively. Genes with paired t-test *P *< 0.05, and fold difference ≥ 14 or ≤-1.4, were identified as differentially expressed genes and lie outside the lines (in red). (D) The heat map shows the level of expression (red: greater up-regulation, blue: greater down-regulation) of each of these genes in all six samples (column 1-3: controls, column 4-6: Day 2, column 6-9: Day 4). An enlargement of the heatmap to the right shows that distinct patterns of gene expression were evident for each of these three datasets.

RNA samples extracted from whole inner ears from adult zebrafish were collected at 2 and 4 dpse (plus controls) and were subjected to microarray analysis. Pairwise comparisons were made between groups such that three gene sets were analyzed: Day 2 (genes regulated at 2 dpse compared to controls), Day 4 (genes regulated at 4 dpse compared to controls), and Day 4: Day 2 (genes regulated at day 4 relative to day 2). Differentially expressed genes with fold changes ≥1.4 and *P*-values ≤ 0.05 were considered to be significantly regulated. A number of significantly regulated transcripts were detected by each pairwise comparison. There were 839 transcripts that were differentially expressed two days following acoustic trauma, 377 transcripts on Day 4, and 505 transcripts on Day 4: Day 2 (Figure 1). To assess the reproducibility within control, Day 2, and Day 4 microarray data, we compared gene expression between triplicate homotypic samples. The mean unnormalized correlation coefficients for all three of these datasets was 0.99, indicating robust consistency between technical replicates. Tables 1, 2, and 3 show the top ten most highly up-regulated and top ten most highly down-regulated genes for each of the following pairwise comparisons- Day 2:Controls, Day 4:Controls, and Day 4:Day 2, respectively. Additional files 1, 2, and 3 list the subpopulations of all genes whose expression was significantly regulated (*P *≤ 0.05), sorted by fold change.

**Table 1:** Classification of the top ten up- and down-regulated transcripts in the ears of *Danio rerio *allowed to recover for two days following acoustic overexposure compared to controls.

Gene ID	Description (gene symbol)	Fold change	P-value	GO Process or Function
AY286447	growth hormone 1 (*gh1)*	64.43	0.0002	Hormone activity
NM_205623	luteinizing hormone beta 1 (*lhbeta1)*	10.10	0.0003	Undetermined
NM_205729	nuclear receptor subfamily 1, group D, member 1 (*nr1d1)*	9.32	0.0002	Transcription
NM_205687	glycoprotein hormones, alpha polypeptide (*cga)*	7.38	0.0083	Hormone activity
BC085676	si:ch211-284a13.1 (*Chst15)*	6.43	0.0003	Undetermined
NM_198815	stearoyl-CoA desaturase (delta-9-desaturase) (*scd)*	5.35	0.0027	Lipid metabolism
NM_181438	proopiomelanocortin (*pomc)*	5.02	0.0015	Signal transduction
AW419856	zebrafish gridded kidney *wu:fj84d10*	4.62	0.0006	Undetermined
NM_212889	zgc:77076 (*zgc:77076)*	4.31	0.0002	Signal transduction
NM_201334	zgc:64065 (*zgc:64065)*	4.07	0.0006	Signal transduction
NM_212439	period homolog 4 (*per4)*	3.86	0.0005	Signal transduction
NM_205676	zgc:77592 (*zgc:77592)*	-3.45	0.0018	Undetermined
NM_001002085	slow-specific troponin C (*stnnc)*	-3.71	0.0004	Calcium ion binding
NM_001004628	zgc:101740 (*zgc:101740)*	-4.66	0.0048	Nucleoside metabolic process
NM_200965	ATPase, Ca++ transporting, cardiac muscle, slow twitch 2a (*atp2a2a)*	-5.31	0.0002	Calcium ion binding
NM_001004112	zgc:92375 (*zgc:92375)*	-5.47	0.0174	Metal ion binding
AI721910	1-acylglycerol-3- phosphate O-acyltransferase 4 (lysophosphatidic acid acyltransferase, delta) (*agpat4)*	-5.57	0.0020	Acyltransferase activity
NM_200516	zgc:66286 (*zgc:66286)*	-29.66	0.0015	Calcium ion binding
AF434191	atrial myosin light chain (*zgc:66286)*	-30.36	0.0009	Undetermined
AF425742	slow muscle myosin heavy chain like (*smyhc1l)*	-36.63	0.0006	Undetermined
NM_194425	major histocompatibility complex, class I, ZE (*mhc1ze)*	-67.68	0.0024	Immunity

**Table 2:** Classification of the top ten up- and down-regulated transcripts in the ears of *Danio rerio *allowed to recover for four days following acoustic overexposure compared to controls.

Gene ID	Description (gene symbol) [homologous organism]	Fold change	P-value	GO Process or Function
NM_205623	luteinizing hormone beta 1 (*lhbeta1)*	10.70	0.0012	Undetermined
AF273879	immunoglobulin heavy variable 2-2 (*ighv2-2)*	10.17	0.0167	Undetermined
NM_205729	nuclear receptor subfamily 1, group d, member 1 (*nr1d1)*	8.33	0.0002	Transcription
BC085676	si:ch211-284a13.1 (*Chst15)*	7.54	0.0022	Undetermined
NM_205687	glycoprotein hormones, alpha polypeptide (*cga)*	7.02	0.0305	Hormone activity
NM_181438	proopiomelanocortin a (*pomca)*	6.95	0.0039	Neuropeptide signaling pathway
AY286447	growth hormone 1 (*gh1)*	5.47	0.0044	Hormone activity
NM_212439	period homolog 1b [Drosophila] (*per1b)*	4.24	0.0010	Transcription
BC054944	transferrin-a (*tfa)*	2.79	0.0003	Iron ion transport
NM_200634	amyloid beta (A4) precursor protein-binding, family B, member 1 interacting protein (*apbb1ip)*	2.68	0.0106	Signal transduction
NM_001002085	slow-specific troponin C (*stnnc)*	-3.26	0.0011	Calcium ion binding
BC045465	matrilin 1 (*matn1)*	-3.27	0.0027	Undetermined
NM_001002119	zgc:86810 (*zgc:86810)*	-3.45	0.0096	Undetermined
NM_131591	actin, alpha 1, skeletal muscle (*acta1)*	-3.45	0.0030	ATP/Nucleotide/Protein binding
NM_201095	zgc:56376 (*zgc:56376)*	-3.61	0.0421	Metal ion binding
NM_200965	ATPase, Ca++ transporting, cardiac muscle, slow twitch 2a (*atp2a2a)*	-4.16	0.0004	Regulation of cation transport, ATP biosynthetic process
NM_205676	zgc:77592 (*zgc:77592)*	-5.50	0.0052	Undetermined
NM_170767	vitellogenin 1 (*vtg1)*	-13.29	0.0067	Lipid transport
AF425742	slow myosin heavy chain 1, like (*smyhc1l)*	-33.26	0.0190	Stress response, contraction
NM_200516	atrial myosin light chain (*zgc:66286)*	-62.03	0.0026	Calcium ion binding

**Table 3:** Classification of the top ten up- and down-regulated transcripts in the ears of *Danio rerio *allowed to recover for four days following acoustic overexposure compared to two days.

Gene ID	Description (gene symbol) [homologous organism]	Fold change	P-value	GO Process or Function
NM_194425	major histocompatibility complex class I ZE gene (*mhc1ze)*	34.36	0.0143	Immunity
NM_001004628	zgc:101740 (*zgc:101740)*	6.82	0.0099	Nucleic acid metabolism
AF273879	immunoglobulin heavy variable 2-2 (*ighv2-2)*	4.54	0.0437	Undetermined
NM_001004587	zgc:92214 (*zgc:92214)*	4.17	0.0116	Metal ion binding
AB062116	heat shock cognate 70-kd protein (*hsp70)*	3.30	0.0029	Response to heat
BC074056	zgc:153863 (*zgc:153863)*	2.61	0.0191	Undetermined
NM_001007378	PTC7 protein phosphatase homolog [S. cerevisiae] (*pptc7)*	2.49	0.0452	Catalytic activity
NM_131108	type I cytokeratin (*cki)*	2.27	0.0099	Structural molecule
NM_001003445	zgc:92533 (*zgc:92533)*	2.25	0.0146	Undetermined
BC059568	zgc:73226 (*zgc:73226)*	2.17	0.0282	Regulation of apoptosis
AI721910	1-acylglycerol-3-phosphate O-acyltransferase 4 (lysophosphatidic acid acyltransferase, delta) (*agpat4)*	-2.73	0.0384	Metabolic process
NM_001007365	troponin I, skeletal, fast 2a.1 (*tnni2a.1)*	-2.73	0.0305	Undetermined
NM_213556	jun B proto-oncogene (*junb)*	-2.81	0.0478	Transcription regualtion
AF500198	fibronectin 1b (*fn1b)*	-2.89	0.0481	Undetermined
NM_212837	potassium voltage-gated channel, subfamily H (eag-related), member 2 (*kcnh2)*	-3.01	0.0411	Signal transduction, transcription, ion transport
NM_200091	signal transducer and activator of transcription 1b (*stat1b)*	-3.11	0.0308	Transcription, transduction
NM_198815	stearoyl-CoA desaturase (delta-9-desaturase) (*scd)*	-3.29	0.0374	Lipid biosynthesis
NM_201334	zgc:64065 (*zgc:64065)*	-3.69	0.0053	Intracellular signalling
NM_170767	vitellogenin 1 (*vtg1)* (replaced by NM_001044897)	-4.25	0.0203	Chemical stimulus response
AY286447	growth hormone 1 (*gh1)*	-11.78	0.0025	Hormone activity

Transcripts showing the greatest regulation on Day 2 compared to control included growth hormone 1 (*gh1*; 64.43-fold), major histocompatibility complex, class I, ZE (*mhc1ze*; -67.68-fold), atrial myosin light chain (zgc:66286; -30.36-fold), and slow muscle myosin heavy chain, like (*smyhc1l*; -36.63-fold). On Day 4, the transcripts showing the greatest fold change included atrial myosin light chain (*zgc:66286*; -62.03-fold) and slow myosin heavy chain 1, like (*smyhc1l*; -33.26-fold). Transcripts showing the greatest fold change in the Day 4: Day 2 dataset included major histocompatibility complex class I ZE (*mhc1ze*; 34.36-fold) and *gh1 *(-11.78-fold). Many of the genes that were significantly regulated on Day 2, were significantly regulated in the opposite direction on Day 4 (Table 4). For example, growth hormone 1 (*gh1*), matrix metalloproteinase 13 (*mmp13*), major histocompatibility complex (MHC) class II integral membrane protein alpha chain 3 (*zgc:92049*), and *junb *were upregulated at Day 2, but downregulated at Day 4 compared to Day 2. In contrast, MHC class I, ZE (*hla-ze*) and MHC class II integral membrane alpha chain (*mhc2a*) were downregulated at Day 2, but upregulated at Day 4 compared to Day 2.

**Table 4:** Representative genes significantly regulated in the *Danio rerio *inner ear at both two and four days post-sound exposure.

GenBank #	Gene name/symbol	Putative biological process	Day 2:Control Fold Change	P-value	Day 4:Day 2 Fold Change	P-value
NM_194425	*hla-ze*	Immunity	-67.68	0.0024	34.36	0.0143
NM_131490	*mhc2a*	Immunity	-2.43	0.0005	4.15	0.0018
NM_001005976	*c1qc*	Immunity	2.93	0.0002	-2.04	0.0149
NM_13169	*zgc:92049*	Immunity	3.09	0.0011	-2.33	0.0249
NM_199730	*dgat1*	Lipid synthesis	2.68	0.0005	-2.18	0.0085
NM_198815	*scd*	Lipid synthesis	5.35	0.0027	-3.29	0.0374
AB055667	similar to *MRPS31*	Mitochondrial protein	-2.13	0.0003	2.01	0.0030
NM_001004628	similar to *Pnp*	Nucleic acid metabolism	-4.66	0.0048	6.82	0.0099
NM_200856	similar to *torsin B*	Protein chaperone	3.78	0.0427	-2.04	0.0287
NM_201503	*mmp13*	Proteolysis	2.39	0.0032	-2.15	0.0161
TC293041	Ubquitin-like protein 2	Proteolysis	4.82	0.0003	-2.77	0.0037
NM_200091	similar to *STAT1*	Signal transduction	3.04	0.012	-3.1	0.0308
NM_212837	*kcnh2*	Signal transduction	3.23	0.0019	-3.01	0.0411
NM_201334	similar to *PLCXD1*	Signal transduction	4.07	0.0006	-3.69	0.0053
AY286447	similar to *GH1*	Signal transduction	64.43	0.0002	-11.78	0.0025
NM_194390	*znfl2*	transcription, apoptosis	-2.48	0.0016	2.14	0.0077
NM_213556	*junb*	Transcription, apoptosis	3.14	0.0005	-2.81	0.0478
AY538257	fibronectin 3	Wound healing	2.78	0.0045	-2.33	0.0146
ENSDART00040691	similar to *F13A1*	Wound healing	5.74	0.002	-3.92	0.0287
TC282441	*Rln3*	Wound healing	8.53	0.0008	-4.97	0.0283
AF434191	atrial myosin light chain (*zgc:66286)*	Calcium ion binding	-30.36	0.0009	------	------
AF425742	*smyhc1l*	Motor activity	-36.63	0.0006	------	------

**Figure 2:**
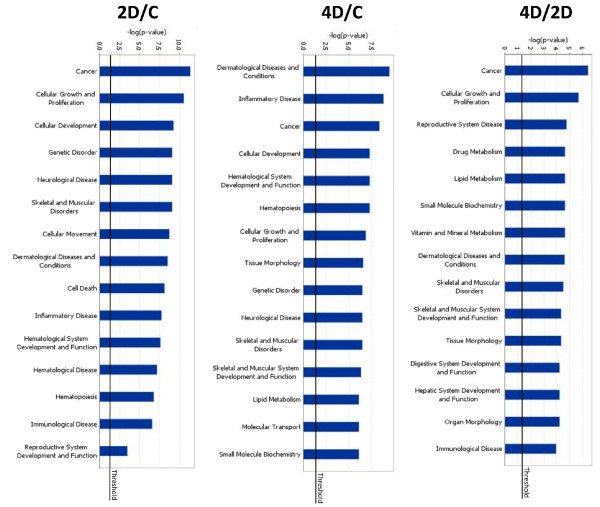
**Functional categories of differential gene expression in the zebrafish ear following acoustic trauma**. The top fifteen functional categories resulting from IPA Functional Analysis for each of the three pairwise comparisons of differentially expressed zebrafish genes. Values of -log (*P*-value) are presented such that more significant (i.e., smaller) *P*-values produce larger bars. *P*-values were calculated using the right-tailed Fisher Exact Test. Comparisons were made between tissues of control and two-days post-trauma, control and four-days post-trauma, and four- and two-days post-trauma zebrafish. Genes associated with functions such as cancer, cellular growth and proliferation, and inflammation are highly regulated in the zebrafish ear following acoustic trauma.

We used *in silico *tools (Ingenuity Pathway Analysis 8.5, Ingenuity Systems, Redwood City, CA) to identify pathways that may be associated with proliferation and hair cell recovery. Functional Analysis of the three sets of transcripts was used to identify significant processes or pathways being affected during the process of auditory cell regeneration in the zebrafish ear. While numerous pathways were significantly regulated, we present only the top 15 categories here (Figure 2). Cancer and cellular growth and proliferation pathways were the most significant functional categories at both 2 and 4 dpse. This is not surprising since previous work showed that cell proliferation peaks at two days following acoustic trauma in the zebrafish ear [33]. Additional files 4, 5, and 6 present the categories and functions of all the genes analyzed for the Day 2:Control, Day 4:Control, and Day 2:Day 4 datasets, respectively.

Cell death pathways were significantly regulated at Day 2 compared to controls. Other processes that were significantly regulated during the first four days following acoustic trauma included cellular development, inflammation, immunology, and dermatological diseases and conditions (Figure 2).

**Figure 3:**
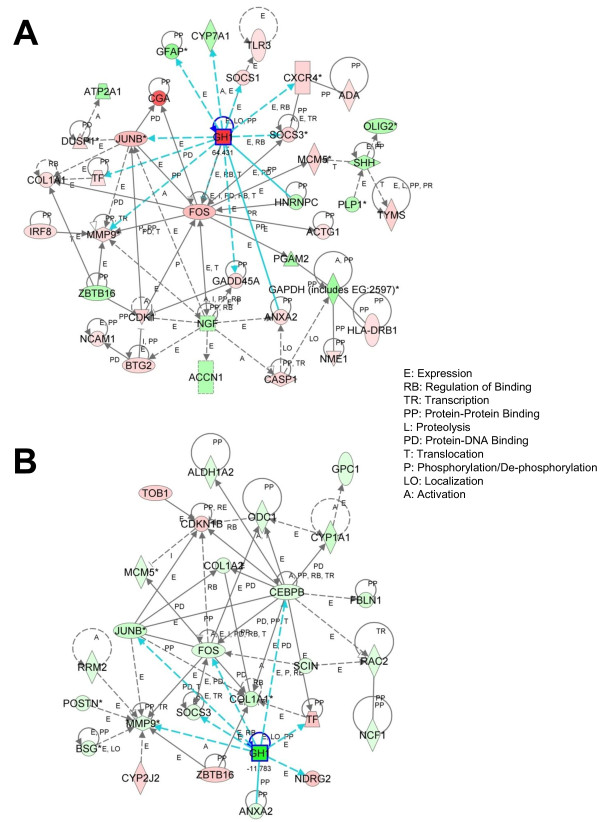
**Functional network analysis of genes related to cell growth in the zebrafish inner ear following acoustic trauma**. Functional network analysis of differential gene expression of genes related to cell growth in the zebrafish inner ear following acoustic trauma. Network (A) compares expression between control and two-days post trauma, while (B) compares four- to two-day post-trauma tissue. Nodes are color-coded according to their *d *score (*red*, overexpression; *green*, underexpression). Solid lines represent direct interactions, while the broken lines indicate indirect relationships. Growth hormone, which has been bolded in both networks, is upregulated two days post-trauma and then is downregulated at four days post-trauma.

Following this initial analysis, we sought to identify gene networks and biochemical pathways of zebrafish homologs of mammalian genes that may be significantly up- or down-regulated in response to acoustic overexposure. Since cancer and cellular proliferation pathways were highly regulated in our dataset, we used functional network analysis to identify specific pathways involved in cellular growth. At Day 2, *gh1 *was the most highly overexpressed gene, but others included *junb*, *fos*, *cga*, *socs1 *and *3*, *cdk1*, and *mmp9 *(Figure 3A). By Day 4, most of these genes were significantly under-expressed (*gh1*, *fos*, *socs3*, *mmp9*, *junb*; Figure 3B).

**Figure 4:**
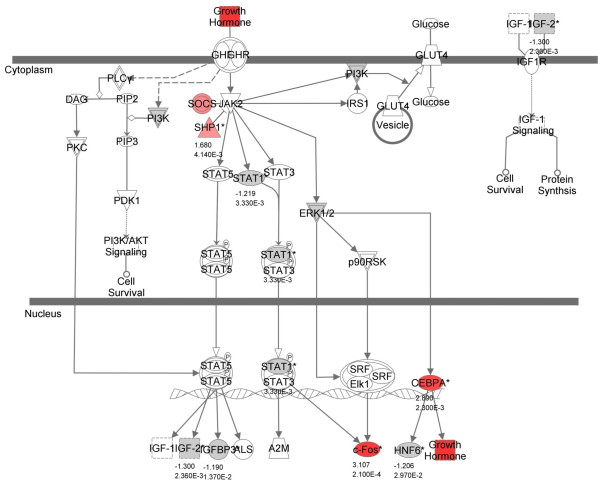
**Canonical pathway analysis reveals growth hormone-related gene regulation**. Canonical pathway analysis showing known growth-hormone related pathways. Red-colored genes are up-regulated and gray-colored genes are down-regulated in the zebrafish microarray data set at two days following acoustic trauma. Numbers below genes represent fold changes and *P*-values.

Zebrafish homologs of mammalian genes reportedly involved in growth hormone pathways were shown to be significantly regulated in the zebrafish microarray dataset (Table 5; Figure 4). The JAK-STAT signaling pathway regulates the production of insulin-like growth factors (IGF). At Day 2, transcripts for *socs *(suppressor of cytokine signaling), *shp1 *(Shatterproof1 mammalian; protein tyrosine phosphatase, non-receptor type 6 zebrafish), and *cebpa *(CCAAT/enhancer binding protein alpha), a regulator of transcription, were upregulated. Both *socs *and *shp1 *are inhibitors of *jak*, and *cebpa *can interact with cyclin-dependent kinases to arrest cell growth [34] so their over-expression could be part of a negative feedback loop associated with a strong GH signal. In contrast, *c-fos *(mammalian; v-Fos FBJ murine osteosarcoma viral oncogene homolog-zebrafish) is also upregulated, and is a proto-oncogene that promotes cell growth and proliferation [35].

**Table 5:** Growth hormone-related transcripts differentially expressed in the ears of *Danio rerio *allowed to recover for two days following acoustic overexposure compared to controls.

Gene ID	Description (gene symbol)	Fold change	P-value	GO Process or Function
AY286447	growth hormone 1 (*gh1)*	64.43	0.0002	Hormone activity
NM_200091	signal transducer and activator of transcription 1b (*stat1b)*	3.04	0.0120	Signal transduction
NM_205569	v-fos FBJ murine osteosarcoma viral oncogene homolog (*fos)*	3.11	0.0002	Transcription
NM_131885	CCAAT/enhancer binding protein (C/EBP), alpha (*cebpa)*	2.89	0.0023	Transcription
NM_213304	*Danio rerio *zgc:77038, suppressor of cytokine signalling 3b (*socs3b)*	1.99	0.0029	Cytokine signalling
NM_001003467	*Danio rerio *zgc:91868, suppressor of cytokine signalling 3b (*socs1)*	1.81	0.0011	Cytokine signalling
NM_199960	*Danio rerio *protein tyrosine phosphatase, non-receptor type 6 (*ptpn6)*	1.68	0.0041	Regulate transduction

### Confirmation of microarray results with real-time quantitative PCR

**Figure 5:**
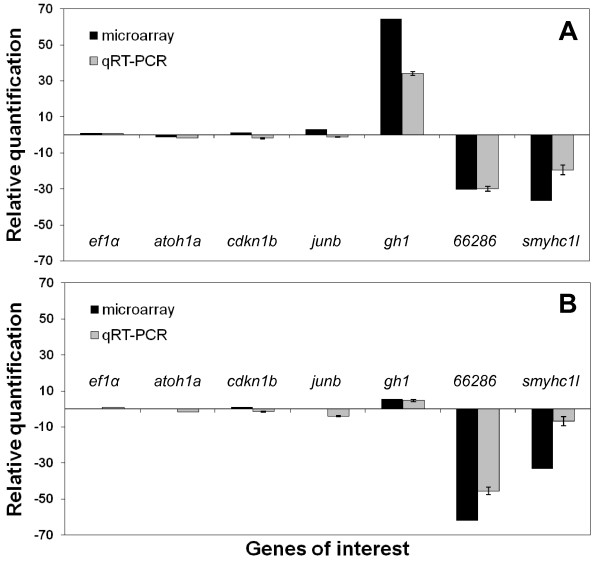
**Validation of microarray data by real-time qPCR**. Mean (± SEM, n = 3) expression of the genes *ef1alpha *(housekeeping gene)*, atoh1a*, *cdkn1b*, *JunB, gh1*, *mlc1*, and *myhc*, as measured by microarray and qPCR. The expression profiles were similar between microarray and qPCR data.

In order to validate the results obtained through microarray analysis, we performed real-time quantitative PCR using probes obtained from custom Taqman Gene Expression Assays (Applied Biosytems) designed against the following target genes: *ef1alpha*, *gh1*, *junb*, *atoh1a*, *rb1*, and *cdkn1b*. Sybr green probes were designed against the following genes: *smyhc1l *, zgc:*66286 *(atrial myosin light chain), *mlc1*, *mhc1ze*, and *ppia*. The pattern of transcript abundance detected for these genes in the array was validated with the aid of real-time PCR (Figure 5). The target genes that were chosen included genes that were up-regulated (*gh1*), down-regulated (*smyhc1l*, *zgc:66286*), and not highly regulated (*atoh1a*, *cdkn1b*, *junb*). In addition, *atoh1a *and *cdkn1b *were chosen since they are known to regulate cell proliferation and hair cell regeneration in the mammalian ear [27-29].

### Effect of growth hormone on cell proliferation in the zebrafish utricle and saccule

**Figure 6:**
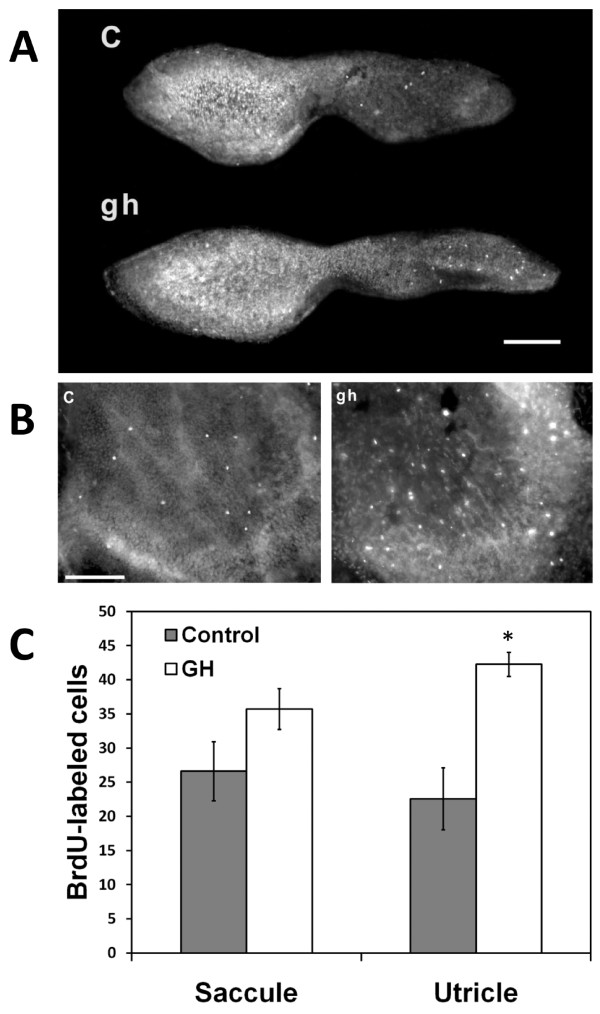
**Growth hormone promotes cell proliferation in the zebrafish inner ear**. A) BrdU-labeled saccules and B) utricles of control (C) and growth hormone-injected (gh) zebrafish. C) Numbers of BrdU-labeled cells increased significantly only in the utricle (*P *≤ 0.001; n = 6) following injection with growth hormone; however, a non-significant but noticeable increase in proliferation occurred in the saccule (*P *= 0.093; n = 6).

We were interested in the effect that overexposure to GH might have in the normal, non-acoustically exposed inner ear, given that *gh1 *levels were so dramatically up-regulated following acoustic trauma. Zebrafish (n = 6) were intraperitoneally injected with salmon GH and allowed to recover 24 h before treatment for bromodeoxyuridine (BrdU) detection through immunofluorescence. Two inner ear end organs were examined: the utricle, which is part of the vestibular system, and the saccule, which is the organ most fully characterized as a sound detector in fishes [36]. Injection with growth hormone resulted in a significant increase in cell proliferation only in the utricle (*P *≤ 0.001), although a deductable increase was also noted in the rostral portion of the saccule (*P *= 0.093; Figure 6).

A small number of proliferating cells was detected in control saccules (mean ± S.E. = 26.6 ± 4.31), which supports previous reports of ongoing proliferation in the adult zebrafish saccule [33,37]. Proliferating cells in control saccules were noted primarily near the rostral tip and near the outer margins, although some BrdU-labeled cells were observed in other portions of the saccule (Figure 6A). Proliferating cells observed in treatment saccules did not show a consistent spatial arrangement in the rostral area. In some saccules, proliferating cells were located primarily near the edges of the rostral area, while in other saccules labeled cells were concentrated in the center of the rostral saccule. The spatial distribution of proliferating cells in the caudal region of the saccule was similar in control and treatment groups. Labeled cells occurred mainly in the outer margins of the macula.

Proliferating cells were also observed in control utricles, primarily near the outer margins of the macula (Figure 6B). Labeled cells in treatment utricles were scattered widely across the entire surface of the utricular macula, with less observable clustering or concentration at the edges than in controls. Proliferating cells in both control and treatment saccules and utricles were observed in multiple cell layers of the sensory epithelia.

## Discussion

Our current approach has been to delineate regulated zebrafish genes in order to provide direction for future investigations into auditory hair cell regeneration in zebrafish and mammals. Distinct patterns of gene expression were evident two and four days after acoustic trauma, suggesting that sound-induced damage in the zebrafish inner ear is a good model system for understanding pathways involved in hair cell regeneration. Transcripts showing the most dramatic regulation over the time course of our study include transcripts encoding growth hormone, major histocompatibility complex, class I, ZE, a light chain myosin, a heavy chain myosin, and a protein similar to atrial myosin light chain (*zgc:66286*).

The short time period within which these transcripts were examined following acoustic trauma coincided with a sharp increase in cell proliferation and partial recovery of hair cell bundle density, which was observed in our previous experiment with zebrafish [33], suggesting that these genes, as well as others listed in the datasets, may play a role in the regulation of cell proliferation and/or cellular repair. Genes associated with transport, kinase activity, transcription factor activity, signal transduction, hormone activity, nucleobase, nucleoside, nucleotide and nucleic acid metabolic process, extracellular region, cellular component, and calcium ion binding were also significantly regulated during this time period. However, a number of genes could not currently be assigned to any process or functional category. The roles of these transcripts during hair cell repair and regeneration remain undetermined. Further work is needed to elucidate the specific roles of many of the genes uncovered in this study.

### A. Role of growth hormone in hair cell regeneration

Mammalian growth hormone (GH) and insulin-like growth factor 1 (Igf1) affect growth in postnatal animals through independent and common pathways [38], influencing final stature [39,40] and facilitating neuron development and survival [41]. No previous study has been published concerning the effect of growth hormone in the inner ear, but other growth-related factors are known to affect hair cell production and survival in mammals. Igf1-null mice exhibit altered inner ear maturation, abnormal innervation of the sensory cells in the organ of Corti, and increased apoptosis of cochlear neurons [42]. Vestibular hair cell proliferation can be stimulated in mammals through exposure to transforming growth factor-alpha and epidermal growth factor [43]. The zebrafish homologs of these genes were not listed among the differentially regulated transcripts in our study, but *gh1 *was dramatically upregulated 64-fold on Day 2 and remained upregulated over five-fold on Day 4, indicating that growth hormone played a prominent role in post-sound exposure recovery of the inner ear of zebrafish. We speculated that the activity of gh1 in the zebrafish might induce proliferation in the ear, since administration of growth hormone can increase cell proliferation in cultured trout leukocytes [44] and increase body mass in zebrafish [45]. To characterize the effect of gh1 on cells of the inner ear, we injected zebrafish with salmon growth hormone. The significant increase we observed in cell proliferation in non-sound-exposed zebrafish inner ear following injection with growth hormone suggests at least three things: 1) growth hormone has the ability to stimulate proliferation in the inner ear of zebrafish, 2) under normal conditions, cells of the utricle, a vestibular organ, are more sensitive to growth hormone-mediated signaling than are cells of the saccule, and 3) that the rostral portion of the saccule may be more sensitive than the caudal portion. The difference in growth hormone sensitivity between the zebrafish utricle and saccule, and the potential difference between the rostral and caudal portions of the saccule, was unexpected but intriguing.

A difference in growth hormone sensitivity may reflect differences in proliferative capacity among the inner ear end organs. The regenerative capacity of hair cells in the fish utricle and lagena has not yet been determined. However, other non-mammalian vertebrates are capable of regenerating hair cells in both the vestibular and auditory portions of the inner ear [3,4,10,14,16], and rates of proliferation differ in vestibular and auditory systems in the absence of damage. For instance, supporting cells in the auditory portion of the chick inner ear, the basilar papilla, are normally quiescent in the absence of a damaging stimulus [46]. Conversely, hair cells in the vestibular organs of chicks have a relatively short life span (approximately 2-6 weeks), undergoing spontaneous apoptosis and replacement though proliferation and differentiation of epithelial supporting cells [47-50]. Hair cells in the mammalian auditory system do not regenerate, but vestibular hair cells exhibit a limited regenerative capacity [51-54]. Low levels of apoptosis occur throughout the development of the zebrafish saccule [37], but no data comparing the rate of apoptosis in the uninjured zebrafish saccule and utricle is currently available. This would be useful in elucidating whether the dissimilar sensitivity of different portions of the zebrafish inner ear to proliferation corresponds with dissimilar rates of apoptosis. In noise-exposed goldfish, apoptosis peaked in the saccule one day before a peak in apoptosis in the lagena, suggesting that patterns of cell damage can vary between different endorgans of the teleost ear [33].

Interestingly, although *gh1 *was up regulated approximately 64-fold at 2 dpse in our experiment with zebrafish, *Gh1 *in the rat cochlea is down-regulated two-fold following temporary threshold shift induced by noise-exposure, and to a smaller extent following permanent threshold shift [55]. Previous studies with mammals have shown that acute or chronic stresses can reduce GH levels in blood serum as well as in the brain [56-58]. While it is possible that exposure to sound produces a different growth hormone regulatory response in mammalian and non-mammalian vertebrates like zebrafish, it is more likely that this is due to differential timing of sound exposure. In the rat cochlea experiment, noise exposures were only for 90 minutes whereas in the current study, zebrafish were exposed for 36 hours followed by a two day recovery period. Thus, in zebrafish there could have been a decrease in GH during the initial stress of the acoustic exposure, followed by a subsequent increase during the recovery and regeneration phase. Future experiments with more time points following acoustic trauma are needed to determine this.

### B. Other transcripts associated with cell proliferation

Other genes that may have been up- or down- regulated in order to enable cell proliferation include signal transducer and activator of transcription 1 (*stat1*), stearoyl-Coa desaturase (*scd*), diacylglycerol O-acyltransferase (*dgat*), and major histocompatablility complex class II (MHC II) genes. The function of *stat1 *may be connected with *gh1*, as it is in mammals, since growth hormone is known to activate signaling pathways that include STAT proteins [Figure 4; [59]]. The STAT activation process is transient and influences a broad range of physiological processes depending on the activating ligands and tissue type [60]. The STATs that are activated by growth hormone exposure can vary by cell type, possibly contributing to the specificity of the growth hormone response [61].

Proteins Scd and Dgat appear to regulate lipid biosynthesis, and possibly phospholipid membrane synthesis. Proliferation depends in part on the ability to incorporate oleate with free long-chain fatty acids in order to form membrane phospholipids [62]. Since the Scd protein synthesizes the oleate necessary for the biosynthesis of membrane phospholipids [63], the *Danio rerio scd *gene may be up-regulated on day two in order to increase production of membrane phospholipids as required by cell proliferation. The protein encoded by *dgat*, another gene up-regulated at 2 dpse and down-regulated between days 2 and 4, also participates in the regulation of membrane lipid synthesis. DGAT proteins interact with diacylglycerols, which are common intermediates for both triacylglycerol and phospholipid synthesis. DGAT tips diacylglycerol toward triacylglycerol synthesis. For instance, *in vitro *overexpression of DGAT1 gene in human lung SV40-transformed fibroblasts reduces synthesis of the membrane phospholipids phosphatidylcholine, phosphatidylethanolamine, and sphingomyelin by 30-40%, and reduces cell growth rate [64]. It is not clear why *dgat *was upregulated on Day 2, given that cell proliferation peaks at this time, but one possibility is that up-regulation of *dgat *occurred as part of the system to regulate proliferation.

Several genes associated with immune function were identified in the microarray. These genes may play roles in cell proliferation following apoptosis. Major Histocompatibility (MHC) class II molecules are found on professional antigen-presenting cells such as macrophages, dendritic cells and B cells. MHC class II molecules are observed in the cochlear cells of adult mice following a damaging event and may promote cell proliferation in the inner ear of organs that possess proliferative capability [65].

Deoxyspergualin, a drug that inhibits *de novo *cell surface expression of MHC class II antigens, blocks cell proliferation in the kidney [66]. Zebrafish MHC complex class II integral membrane alpha chain gene (*mhc2a*) was significantly regulated on 2 dpse and between days 2 and 4 dpse. Even more notable is MHC complex, class I, ZE (*mhc1ze*), which was down-regulated more than 67-fold on 2 dpse, but was not significantly regulated by 4 dpse. At this time, the function of mhc1ze has not been determined, but since MHC class I proteins are involved in antigen presentation on nearly all cell types in mammals, it seems probable that mhc1ze functions similarly in zebrafish. Antibodies that bind human MHC Class I molecules (HLA) and prevent them from presenting antigens induce increased proliferation of airway epithelial cells [67]. Down-regulating *mhc1ze *in zebrafish may have a similar effect, encouraging proliferation by the reduction of antigen presentation.

It is not surprising that genes related to immune function were regulated following acoustic trauma since macrophages, a type of leukocyte, are recruited to sites of damage and may be involved in initiating wound healing and repair [68]. Within hours of trauma to hair cell sensory epithelium, macrophages and other leukocytes are recruited to the area of damage. This has been reported in the lateral line of amphibians [69] and zebrafish [70], avian inner ear sensory epithelia [71-73], and the mammalian organ of Corti [74]. Macrophages recognize and destroy cells undergoing apoptosis via phagocytosis [75] and may secrete substances such as growth factors that could affect cell proliferation and other functions [68,76]. It has long been recognized that there is an interaction between the endocrine and immune systems in mammals. This appears to be true in fishes as well, and GH may be an important mediator between the two systems. For example, plasma GH levels and phagocytic activity are positively correlated in brown trout (*Salmo trutta*) during sea-water transfer [77,78], and GH causes proliferation in leukocyte cultures of chum salmon, *Onchorynchus keta *[79]. Reciprocal effects are also evident. Stress induces a rapid decrease of plasma GH levels in several fish species [80-82].

Another group of proteins that were highly regulated in our dataset were myosins. The most highly regulated were atrial myosin light chain (*zgc:66286*, -30 fold on Day 2, and -62 fold on Day 4) and slow muscle myosin heavy chain, like (*smyhc1l*, -36 fold on Day 2, and - 33 fold on Day 4). Mutations in non-muscle myosins MYH9, MYH14 and myosin VIIa have been implicated in deafness in mammals [83-85]. Myosins are a large superfamily with many shared domains among the members and are important regulators of the actin cytoskeleton, a prominent component of hair cell bundles. A large number of different myosins are expressed in developing neurons and sensory cells, helping to carry out a range of functions including morphogenesis, axonal transport, and synaptic and sensory functions [reviewed in [86]], although the functions of many myosins are not known [87].

It is not clear why *smyhc1l *was down-regulated following acoustic trauma; however, *smyhc1l *may play a role in the regulation of immune response in the inner ear. Smyhc1l is a TMPIT-like protein, which is induced by TNF-alpha [88]. Since TNF-alpha is a cytokine involved in inducing immune response, apoptosis and inflammation [89], it is reasonable to assume that the down-regulation that we see in *smyhc1l *may be associated with the down-regulation in TNF-alpha and other cytokines that one would expect during the recovery from inflammation. In support of this, a number of genes that are negative regulators of immune response were up-regulated two days post-trauma, including TCF family B cell activation factor (TC277656), C1q tnf1 protein (TC276192), and complement C1q tumor necrosis factor-related protein 4 precursor (TC298139; Additional file 1).

Atrial myosin light chain (*zgc:66286*) possesses an EF-hand domain [88]. EF hands are a superfamily of calcium sensors and calcium signal modulators. Calcium-binding proteins such as calretinin, calmodulin, and parvalbumin have been used as markers for inner ear ganglion neurons and hair cells [90-94]. Calmodulin is known to mediate inflammation, apoptosis, immune response, and cell cycling [95,96], but it is unclear at this point if the calcium-binding properties of atrial myosin light chain are serving similar roles in the zebrafish inner ear.

### C. Genes associated with induced hair cell regeneration in mammals

Zebrafish homologs of genes that have been used to induce hair cell regeneration in mammals, specifically, cyclin-dependent kinase inhibitor *p27*(*kip1*)/*cdkn1b*, retinoblastoma1 (*rb1*), and atonal homolog 1 (*atoh1*) were found to be regulated at the *P*-value 0.05 level, but not at fold changes ≥1.4. Two days following sound exposure, *cdkn1b *was down-regulated slightly (-1.12-fold, see Additional file 1), while *cdkn1b *and *rb1*, both suppressors of cellular proliferation, showed up-regulation (1.60- and 1.36-fold, respectively) at 4 dpse following the peak in proliferation (Additional file 3). A similar pattern was evident for *atoh*1, which was down-regulated at 2 dpse (-1.20 fold) and up-regulated at 4 dpse (1.24 fold). Thus, more work will need to be done to rule them out as players in the process of proliferation and differentiation of zebrafish hair cells.

In this study, we used RNA isolated from whole ear tissue because of the very small size of the sensory epithelium of the zebrafish inner ear. RNA collected only from sensory maculae or specific cell types may reveal significant regulation of low-abundance transcripts that was not detectable in whole ear samples. Additionally, regulation of proteins, which would not be detected via microarray, likely affects cellular processes during regeneration in the inner ear. Levels of existing p27^Kip1 ^protein may have been altered by ubiquitinylation in order to allow proliferation to occur. Analysis of p27^Kip1 ^protein alteration in the sound-exposed inner ear will be necessary to ascertain whether p27^Kip1 ^protein regulation plays a significant role in naturally occurring hair cell regeneration in the zebrafish. Interestingly, p27 ^Kip1 ^was not found to be a part of the zebrafish hair cell transcriptome [97], although it is a supporting cell marker in the mammalian organ of Corti that inhibits cell cycle progression [98]. Knock-out mice without this gene exhibit cell proliferation in the organ of Corti [27].

The gene *rb1*, was also not significantly regulated in this study at the 1.4 fold cut-off level, but since Rb1 function is regulated by phosphorylation, significant changes in overall transcription levels may not be necessary to promote proliferation. Hypophosphorylated Rb1 is an active proliferation repressor, but Rb1 loses all repression function if sufficiently phosphorylated [99]. The phosphorylation state of pRb following noise exposure will need to be delineated to determine whether pRb is an active regulator of cell proliferation in the zebrafish inner ear.

Similarly, regulation of zebrafish *atoh1a*, homolog of the hair cell differentiation gene *Atoh1/Math1*, was weak at 2 and 4 dpse in our study. Atoh1a is a key regulator of differentiation of precursor cells that become hair cells in mice [24,25]. *Atoh1a *and *b *are also necessary for hair cell differentiation in zebrafish [26]. The time points investigated in this study may have been too early in the recovery process for Atoh1 detection, as Atoh1 only promotes the final stages of hair cell development [24,100] and may have peaked in the majority of regenerating hair cells later than 4 dpse.

### D. Hair cell genes

Comparison of our microarray dataset with the zebrafish hair cell transcriptome [97] revealed common hair cell genes. We identified significant regulation in zebrafish hair cell genes encoding proteins such as creatine kinase, alpha-tubulin, keratin 8, and v-fos FBJ murine osteosarcoma viral oncogene homolog. Two zebrafish genes encoding creatine kinase (creatine kinase, muscle (*ckm*) and creatine kinase, mitochondrial 2 (*ckmt2*)) were significantly regulated in our microarray dataset. Muscular creatine kinase performs a variety of functions, even in non-muscle tissues and cells [101]. In the inner ear, creatine kinase (or its mitochondrial creatine kinase isoform) is required to maintain energy homeostasis through ATP delivery to plasma-membrane Ca^2+^-ATPase isoform 2 (Pcma2), an ion pump required for normal sensory transduction in stereocilia of mammals and birds [102]. In the avian utricle, creatine kinase B is primarily localized in hair cells, and creatine kinase/mitochondrial creatine kinase isoform double knockout mice exhibit elevated hearing thresholds of 20-30 dB at 8 and 16 kHz [103].

Significant regulation of transcripts encoding zebrafish inner ear structural proteins was noted in our study. Alpha-tubulin and beta-tubulin dimers are components of all polymerized microtubules. Strong labeling for alpha tubulin is seen in sensory and supporting cells of the guinea pig inner ear [104]. Keratin 8 is one of the major intermediate filaments, which provide structural support throughout many tissue systems. Keratin 8 is thought to confer resistance to apoptosis induced by Fas ligand or TNF family receptors [105], both of which are implicated in cisplatin- and ethacrynic acid-induced apoptosis of hair cells in chinchillas [106].

V-fos genes (the viral homologue of c-fos genes) are highly inducible in response to a variety of growth factors and differentiation-specific inducers, and can induce bone tumors in mice [107]. Members of the fos and jun protein families can combine to form a complex called activating protein-1 (AP-1). AP-1 induction by the hair cell-toxic antibiotic gentamicin is transient and occurs exclusively in hair cells in rat organ of Corti explants [108]. Inhibitors of the upstream pathway for AP-1 rescue hair cells [109]. It should be noted that the up-regulation of some genes found in our microarray data, such as *c-fos*, are indicative of a general neuronal stress response in fishes [110], and acoustic stimuli can induce a short-term stress response in goldfish [111]. Thus, it is unclear if such regulation is the response from hair cell damage or auditory nerve overstimulation, but it should not be indicative of neuronal changes in brain activity since our samples only contained ear tissue.

Some of the regulated genes in the current study are similar to genes highly regulated in the hair cells of other model organisms as well. Avian utricular hair cell genes include parvalbumin, which serves as a mobile Ca^2+ ^buffer in the avian inner ear, alpha-tubulin, creatine kinase, heat shock protein 90 (HSP90), and an isoform of Ca^2+ ^transporting ATPase [103]. Additionally, POU domain transcription factors, thyroid hormone receptor [112], heat shock proteins [113], and collagen IV alpha chain 4 [114] have been noted in mammalian hair cells. Bcl-2, another regulated gene in our dataset, is believed to play an essential role in prevention of sensory cell death in guinea pigs [115]. Thus, a number of the gene products that were regulated in the zebrafish ear following acoustic trauma have been found in hair cells or have been found to regulate hair cells.

## Conclusions

Microarray analysis of RNA from acoustically overexposed zebrafish inner ears revealed that genes involved in multiple processes were significantly regulated, including those involved with cell proliferation, apoptosis, wound healing, signal transduction, transcription, growth, immunity, and hair cells. Some of these genes are prospective targets for manipulating cell proliferation and/or improving hair cell protection during or following noise exposure. Genes previously identified in the hair cells of zebrafish, and homologs of avian and mammalian hair cell genes were also noted. More work will be needed to determine the functions of these and other genes identified in acoustically-overexposed zebrafish. Although a clear candidate for regulation of mammalian auditory hair cell regeneration has not been identified in this study, the data point to possible additional targets of investigation and suggest that hair cell proliferation may be accelerated through treatment with growth hormone.

## Methods

### Experimental animals

Adult breeder zebrafish (*Danio rerio*) were obtained from Segrest Farms (Gibsonton, FL) and maintained in 170-L flow-through aquaria under conditions of constant temperature (25°C) and a 12-h light/12-h dark schedule. Fish total lengths ranged from 36 to 44 mm. All work was done under the supervision of the Institutional Animal Care and Use Committee of Western Kentucky University.

### Sound exposure

Adult zebrafish were randomly assigned to treatment and control groups without bias for weight or length or sex. Forty zebrafish were exposed to a 100 Hz tone at 179 dB re 1 μPa RMS. The sound was generated by a B&K Precision function generator (4017A) connected to a 5.3 amp/200 watt Audiosource monoblock amplifier and University Sound UW-30 underwater speaker placed in a 19-L sound exposure chamber. Fish were exposed for 36 hours at 24.5-25°C, and then 20 fish were moved to a recovery tank for two days and the remaining 20 fish were placed in another tank for four days. Controls (n = 20) were placed in the sound exposure chamber for the same time and temperature with the sound generator turned off.

### RNA isolation and preparation

RNA samples were obtained from the inner ears of the three groups of 18 to 20 fish each (controls, 2 dpse, 4 dpse). One group served as non-sound-exposed controls, and the remaining two groups were exposed to the acoustic stimulus and allowed to recover for 2 or 4 days. The day 2 time point was selected in order to investigate gene expression during proliferation, which had been shown to peak at 2 dpse, and 4 dpse was chosen since it represented a post-proliferative phase [33]. Additionally, it was hoped that genes strongly associated with hair cells would be significantly regulated at this time point as proliferating cells potentially differentiated into replacement hair cells.

Fish were sacrificed one at a time with an overdose of MS-222, their heads were removed, and both whole ears (saccule, lagena, utricle and semi-circular canals) were immediately dissected out while being completely submerged in RNAlater (Ambion, Austin, TX), as preliminary work indicated that either the small size of the saccule, or the length of time needed to separate it from the inner ear, resulted in low RNA yield. Ears were then placed in sterile Eppendorf tubes and flash frozen in liquid nitrogen. Three to four hours were required to dissect all the fish in one group. Although each fish was dissected quickly, the ears were not contaminated with surrounding tissue other than perhaps residual parts of the auditory nerve. Once all the ears for a sample were collected, the tissue was pooled and homogenized with a Kontes Pellet Pestle Microgrinder and sterile disposable pestles (Kontes, Vineland, NJ), then processed for RNA isolation using the RNeasy Lipid Tissue Mini Kit (Qiagen, Valencia, CA). RNA quality was checked with the aid of an Agilent 2100 Bioanalyzer (Agilent, Wilmington, DE). For this project, sharp ribosomal RNA bands were evident with an RNA integrity number greater than 7.0. 300 ng total RNA was used to generate fluorescent cRNA with the aid of Low RNA Input Linear Amplification kit with one-color (Agilent, Wilmington, DE). Briefly, this kit uses a T7 promoter primer to synthesize cDNA and T7 RNA polymerase to synthesize cRNA, which simultaneously amplifies the target material and incorporates cyanine 3-labeled CTP (Cy3). The labeled cRNA was purified by using the RNeasy Mini Elute kit (Qiagen, Valencia, CA). The yield and incorporation efficiency were measured on a spectrophotometer (NanoDrop Technologies). The yield for this project was greater than 1.5 μg, and the specific activity was greater than 9.0 pmol Cy3 per μg cRNA.

### Microarray

1.65 μg of each labeled cRNA sample was fragmented at 60°C for 30 min (Agilent Gene Expression Hybridization kit) and then hybridized to Agilent Zebrafish (*Danio rerio*) oligonucleotide arrays (Agilent Unrestricted AMADID Release GE 4 × 44K, 60-mer oligonucleotides; G2519F; V1: 015064) at 65 °C for 17 hours. This microarray has 21,000 *D. rerio *probes, replicated twice. Three technical replicates were hybridized for each of the three time points (control, Day 2, and Day 4), with one replicate of each time point on each of the three 4-array plates processed. After hybridization, the microarray slides were washed with Agilent gene expression wash buffers. The slides were scanned with the aid of an Agilent microarray scanner (G2565BA) with a setting for one-color using the green channel and 5 μm resolution. The one-color microarray images (.tif) were extracted with the aid of Feature Extraction software (v 9.5.1, Agilent). Raw and processed gene expression data were deposited in NCBI's Gene Expression Omnibus (GEO, http://www.ncbi.nlm.nih.gov/geo/ webcite, GEO Series accession number GSE29669).

### Quantitative Real Time PCR

Validation of the results obtained through microarray analysis was performed via quantitative PCR on the same RNA samples used for the microarrays. Probes were obtained from custom Taqman Gene Expression Assays (Applied Biosytems) designed against the following target genes: elongation factor 1-alpha, *ef1alpha *(NM_131263); growth hormone, *gh1 *(AY286447); jun B proto-oncogene, *junb *(NM_213556); atonal homolog 1a, *atoh1a *(NM_131091); retinoblastoma 1, *rb1 *(BC154730); and cyclin-dependent kinase inhibitor 1b, *cdkn1b *(NM_212792). Sybr green probes were designed against the following genes: Slow myosin heavy chain, *myhc5 *(AF425742); atrial myosin light chain, *zgc:66286 *(AF434191); *Danio rerio *major histocompatibility complex class I, *mhc1ze *(NM_194425); peptidylprolyl isomerase A (cyclophilin A), *ppia *(NM_212758). Primer sequences are presented in Table 6.

**Table 6:** Primer sequences of *Danio rerio *genes used in qRT-PCR for validation of microarray data.

Symbol	ID	Direction	Primer Sequence	PCR
*ef1alpa*	NM_131263	forward	CGACAAGAGAACCATCGAGAAGTT	Taqman
		reverse	CCCAGGCGTACTTGAAGGA	Taqman

*gh1*	AY286447	forward	Applied Biosystems Assay ID:	Taqman
		reverse	Dr03128643_m1	Taqman

*junb*	NM_213556	forward	Applied Biosystems Assay ID:	Taqman
		reverse	Dr03204057_s1	Taqman

*atoh1a*	NM_131091	forward	GGCAGATGAGGGCAGACA	Taqman
		reverse	CCTCTGTTTCTGCACGACGTT	Taqman

*rb1*	BC154730	forward	GCCCCTCCATCACAACCA	Taqman
		reverse	GGCTCGGCCTCCATTACAG	Taqman

*cdkn1b*	NM_212792	forward	GAGAGCCGAGGAAAAGAAGCT	Taqman
		reverse	GCGAGCGTTTGCTTTGACA	Taqman

*smyhc1l*	AF425742	forward	TGAGCAACTTGGTGAGAGTGGGAA	Sybr green
		reverse	TCAGCTTCCTCCAGAGCAGTTTGT	Sybr green

*zgc:66286*	AF434191	forward	TTCCTGCCAATGCATCAGCACAT	Sybr green
		reverse	CCGTTGCCCTCTTTGTCAAACACT	Sybr green

*mhc1ze*	NM_194425	forward	AGAGTGTGTGGACTGGCTCAACAA	Sybr green
		reverse	AGAATCCAGTGGCCAGACAAGTGA	Sybr green

*ppia*	NM_212758	forward	AGAATTTCAGGCAGTTGTGCACGG	Sybr green
		reverse	TGTGGTTTGTGAAGTCACCTCCCT	Sybr green

Complementary DNA was generated starting with 100 ng of RNA template per reaction using Multiscribe Reverse Transcriptase (Applied Biosystems). Real-time PCR was performed using Taqman or Sybr Gene Expression Master Mixes. Samples were placed in an ABI PRISM 7300 Real-time PCR System (Applied Biosystems) and thermal cycling was initiated at 95°C for 10 min, followed by 40 cycles of denaturing at 95°C for 15 s with annealing at 60°C. Each gene expression was repeated in three independent reactions. Each target gene was normalized relative to endogenous control genes for cyclophilin and ef1-alpha. Single band specificity was verified.

### Immunohistochemistry

Fish averaging 4.7 cm total length and 0.66 g were randomly assigned to treatment and control groups without bias for weight or length. Treatment fish were injected with 10 μg salmon GH/g body weight, while controls were injected with a phosphate buffer solution. Both groups (n = 6/group) were then allowed to recover for 24 h at 25 μC. Cell proliferation in saccules of these fish was then quantified through visualization of cells labeled for BrdU, which is a synthetic thymidine analog that is incorporated into cellular DNA during S-phase. BrdU (Sigma-Aldrich, St. Louis, MO) was dissolved into normal Ringer's solution at a concentration of 5 mg BrdU/ml. Fish were injected intraperitoneally with 0.02 ml BrdU/Ringer's solution and allowed to recover for 4 h. The fish were then euthanized with an overdose of MS-222. The heads were removed and placed in 4% paraformaldehyde overnight at 4°C. The heads were then rinsed 4 × 10 min in 0.1 M PBS and the inner ears dissected out under a stereomicroscope.

The saccules and utricles were isolated from the ears and excess tissue was trimmed away to allow the maculae to lie flat. The maculae were bathed in 1N HCL for one hour at 37°C to denature DNA, 0.1 M borate buffer (pH 8.5) for 10 min to neutralize tissue pH, and washed 3 × 10 min in PBS. Maculae were incubated overnight at 4°C in mouse monoclonal anti-BrdU antibody (Invitrogen, Carlsbad, CA) diluted to 1:100 in 1% BSA/0.5% Triton X-100/PBS. Maculae were then washed 3 × 10 min and incubated for 30 min at room temperature in 1:500 Alexa Fluor 568-conjugated rabbit anti-mouse antibody (Invitrogen) in PBS. Maculae were again washed 3 × 10 min in PBS and mounted with Prolong Gold Antifade reagent with DAPI (Invitrogen). The slides were cover-slipped and viewed under an Zeiss Axioplan 2 epifluorescent microscope with rhodamine and DAPI filters. Images were captured with an AxioCam MRm camera and analyzed with Zeiss Axiovision 4.4 software. Alexa Fluor 568-labeled cells were counted for each whole saccule and utricle to quantify cell proliferation.

### Data analysis

The raw data files generated by the microarray procedure were imported into GeneSpring (GX 7.3) and the data were normalized and analyzed. GeneSpring generated an average value of the three replicates of each gene. Data was transformed to bring any negative value to 0.01. Normalization was performed using a per-chip 50th percentile method that normalizes each chip on its median, allowing comparison among chips. Then a per-gene on median normalization was performed, which normalized the expression of every gene on its median among samples. The differentially expressed genes of significance were evaluated with the aid of Volcano Plots (*P*-value versus fold change; Figure 1). Pairwise comparison of the experimental and control groups used the data derived from the Volcano Plots. Pairwise comparisons were also performed between the two treatment groups. Differentially expressed genes with *P*-values≤0.05 and fold changes ≥ 1.4 were determined to be significantly regulated. The Benjamini and Hochberg False Discovery rate was used for test correction.

Gene networking analyses were performed using Ingenuity Pathways Analysis (IPA; Ingenuity Systems). Biological processes and molecular functions were identified for significantly regulated transcripts via Gene Ontology (GO; http://www.geneontology.org). The processes and functions listed should be considered putative, as many of the genes are currently assigned to GO categories based on electronic annotation or inferences from expression pattern.

The effect of growth hormone injection on cell proliferation in the zebrafish utricle and saccules was tested using a separate one-way analysis of variance for the utricles and saccule. Preliminary analyses showed no statistical differences between right and left utricles and saccules in terms of numbers of BrdU-labeled cells, so data from both ears were pooled for analysis.

## Authors' contributions

JBS performed dissections and RNA extraction of the zebrafish ears, analyzed gene functions, and wrote a majority of the initial manuscript. HS performed Ingenuity Pathway Analysis to examine networks of gene regulation and edited the manuscript. WTP performed RT-PCR confirmation of the microarray data and wrote parts of the paper. NGFC and XL performed the microarray analysis. MES conceived the study, did statistical analysis, and wrote and edited much of the paper. MES, XL and HS prepared the figures. All authors read and approved the final manuscript.

## Supplementary Material

Additional file 1 **Differential gene expression at two days post-trauma compared to controls**. This file presents all significantly regulated (*P *< 0.05) genes in zebrafish ears two days following acoustic trauma compared to controls, sorted by fold change. Following IPA notation, upregulated and downregulated genes with > 1.4 fold changes are labeled in red and green, respectively. Click here for file

Additional file 2 **Differential gene expression at four days post-trauma compared to controls**. This file presents all significantly regulated (*P *< 0.05) genes in zebrafish ears four days following acoustic trauma compared to controls, sorted by fold change. Following IPA notation, upregulated and downregulated genes with > 1.4 fold changes are labeled in red and green, respectively. Click here for file

Additional file 3 **Differential gene expression at four days post-trauma compared to day two**. This file presents all significantly regulated (*P *< 0.05) genes in zebrafish ears four days following acoustic trauma compared to two days post-trauma, sorted by fold change. Following IPA notation, upregulated and downregulated genes with > 1.4 fold changes are labeled in red and green, respectively. Click here for file

Additional file 4 **Significantly regulated functions at two days post-trauma**. This file displays the categories and functions of significantly regulated genes in zebrafish ears two days following acoustic trauma compared to controls. In addition, specific molecules being regulating in each functional annotation are provided. Click here for file

Additional file 5 **Significantly regulated functions at four days post-trauma**. This file displays the categories and functions of significantly regulated genes in zebrafish ears four days following acoustic trauma compared to controls. In addition, specific molecules being regulating in each functional annotation are provided. Click here for file

Additional file 6 **Significantly regulated functions at four days post-trauma compared to day two**. This file displays the categories and functions of significantly regulated genes in zebrafish ears four days following acoustic trauma compared to day two. In addition, specific molecules being regulating in each functional annotation are provided. Click here for file
